# Molecular and epidemiological updates on cystic echinococcosis infecting water buffaloes from Egypt

**DOI:** 10.14202/vetworld.2016.1355-1363

**Published:** 2016-12-04

**Authors:** Ibrahim Abbas

**Affiliations:** Department of Parasitology, Faculty of Veterinary Medicine, Mansoura University, Egypt

**Keywords:** buffalo, *Echinococcus*, Egypt, genotype, prevalence

## Abstract

**Aim:**

Cystic echinococcosis (CE) represents a serious parasitic disease at both animal and public health levels. The majority of reports negated the CE infection in buffaloes from Egypt; however, one study illustrated their infection with G6 genotype (camel strain). The present work contributed to update the epidemiological and molecular knowledge about CE infecting this economically important animal for better understanding of its role in maintaining the *Echinococcus* life cycle.

**Materials and Methods:**

A total of 120 slaughtered water buffaloes at Mansoura abattoir, Dakahlia province, Egypt, were inspected for the existence of hydatid cysts. Cysts location and fertility were examined. Five out of 27 revealed cysts were tested molecularly using both cytochrome C oxidase subunit 1 and nicotinamide adenine dinucleotide hydrogen subunit 1 (nadh1) genes.

**Results:**

Low prevalence (4.2%) as well as considerably low fertility rate (14.8%) of buffaloes CE was noted. G1 genotype (common sheep strain) was revealed from the five examined cysts. At the level of nadh1 partial sequences, a globally singleton G1 haplotype was reported.

**Conclusion:**

This the first report about the G1 infection in buffaloes from Egypt. This study proposed the minimized role of this animal in echinococcosis transmission. These findings could provide preliminary data for the local control of this disease.

## Introduction

Cystic echinococcosis (CE) is an important parasitic infection which caused by the larval stages (hydatid cyst) of the genus *Echinococcus* (a dog tapeworm) [1]. This parasite has a two-host life cycle in which canines serve as definitive hosts, while the intermediate host role is played by the domestic and wild ungulates [2]. Transmission of infection is initiated by ingestion either of *Echinococcus* eggs by the intermediate host or the larval stage by the definitive host.

CE constitutes a serious animal health concern in many rural areas of the world altogether with its public health hazards. From the economic perspective, CE causes important economic losses originated from decreased productivity and viscera condemnation in livestock species. In Ismailia city abattoir (a small Egyptian abattoir), the estimated annual loss in livestock due to CE was 36,480 Egyptian pounds which represented by total or partial condemnation of 1216 kg of meat and offal [3].

Buffaloes are widely reared in Egypt. Economically, it considered the second important animal, providing meat, milk, skin, and hides. In Egypt, Dyab *et al*. [4] did not detect CE infection in buffaloes, while Haridy *et al*. [5] recorded 6.4% infection rate.

Recently, the taxonomic revisions of *Echinococcus* illustrated its complexity into a number of species and genotypes exhibiting a marked genetic variability, based on mitochondrial DNA analysis. Studies have identified 10 distinct genotypes (G1-G10) within four species of the *Echinococcus granulosus* complex [6]. These include *E. granulosus sensu lato* (G1-G3 cluster), *Echinococcus equinus* (G4), *Echinococcus ortleppi* (G5), and *Echinococcus canadensis* (G6-G10). The genotypes are classified into two sheep strains (G1 and G2), two bovid strains (G3 buffalo and G5 cattle), a horse strain (G4), a camel strain (G6), two pig strains (G7 and G9), and finally two cervid strains (G8 and G10). Moreover, a lion strain, *Echinococcus felidis*, was reported. These variants are broadly distributed geographically and have a wide range of host specificity.

Reports about the genotypes of CE infecting buffaloes are considerably low due to the scarcity number of countries in which buffaloes can accommodate to living. The majority of studies illustrated the susceptibility of this animal species to the G1-3 cluster [7,8] although cattle strain (G5) was reported in India [9,10].

Molecular studies on hydatid cyst isolates from the Egyptian animals and human illustrated the presence of G1, G4, G5, and G6, which considered the most prevalent genotype [11-13]. Previously, 2 buffalo samples, obtained from Cairo, were genotyped as G6 (camel strain) [13]. Herein, we examine the slaughtered water buffaloes at Dakahlia province for hydatid cysts to update the CE epidemiology and to clarify if there are genotypes other than G6 infecting water buffaloes from Egypt.

## Materials and Methods

### Ethical approval

This study was conducted after getting approval from both Scientific and Animal Ethics Committees of Faculty of Veterinary Medicine, Mansoura University, Egypt.

### Samples collection and DNA isolation

A 120 water buffaloes slaughtered at Mansoura Abattoir, Dakahlia Province, Egypt, were examined for the presence of hydatid cysts. Cysts were dissected out and washed with phosphate buffer saline (PBS). Cysts fertility was assessed by examining the hydatid fluid and the germinal layer for protoscolices. The germinal layer, as well as the protoscolices, was harvested. Both were washed 3 times in PBS (pH 7.2) and stored at −20°C until used. Total DNA of protoscolices and/or germinal layers was extracted using the NucleoSpin^®^ Tissue kit (Macherey-Nagel, Düren, Germany), according to the manufacturer’s instructions.

### Molecular analysis

Two mitochondrial genes were amplified by polymerase chain reaction (PCR): The cytochrome C oxidase subunit 1 (cox1) gene [14] and the nicotinamide adenine dinucleotide hydrogen subunit 1 (nadh1) gene [15]. Primers COI 1 (forward) 50-TTTTTTGGCCATCCTGAGGTTTAT-30 and COI 2 (reverse) 50-TAACGACATAA CATAATGA AAATG-30 were used to amplify the cox1 gene by 30 cycles. Each cycle consisted of denaturation at 94°C 30 s, annealing at 55°C 30 s and elongation at 72°C 30 s followed by a final extension at 72°C 7 min. Primers NADH 1 (forward) 50-AGTCTCGTAAGGGCCCTAACA-30 and NADH 2 (reverse) 50-CCCGCTGACC AACTCT CTTTC-30 were used to amplify the nadh1 gene by 35 cycles. Each cycle consisted of denaturation at 94°C 30 s, annealing at 53°C 30 s, and elongation at 72°C 30 s followed by a final extension at 72°C 7 min. A negative control (without genomic DNA) was included in the study.

**Figure-1:**
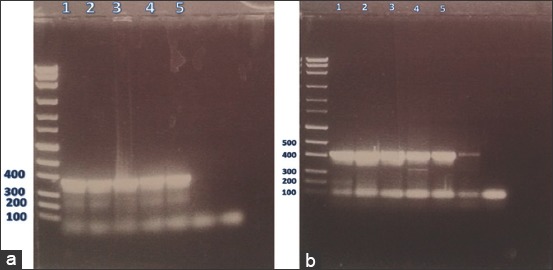
(a-b) Polymerase chain reaction results of cytochrome C oxidase subunit 1 (a) and nicotinamide adenine dinucleotide hydrogen 1 (b) genes on 1% agarose gel stained with ethidium bromide. From left to right: DNA marker, four fertile lung cysts (1-4) then one sterile liver cyst (5) followed by caseated lung cyst failed to be amplified and the negative control.

PCR amplification was carried out in 35 µL final mixture containing 2 µL of template DNA, 1 µL (25 µM) of each primer, 0.7 µL (10 mM) deoxynucleotide triphosphate mix, 3.5 µL of Taq buffer (×10), 0.35 µL Taq polymerase (5 Prime Perfect Taq™), and 26.45 µL nuclease free water. PCR products were separated on agarose gels (1%) stained with ethidium bromide (Figure-1). Gel bands were cut out, and DNA was purified using QIAquick gel extraction kits (Qiagen, Germany) following the manufacturer’s instruction. The purified DNA was commercially sequenced in both directions. Genotypes identification and the reference sequences (Tables-1 and 2) were achieved using GenBank with the BLAST system. Bioedit software was used to align the different sequences, while the phylogenetic analysis was initiated using the neighbor-joining method of the software Mega (version 6) with Kimura 2-parameter model.

**Table-1:** Partial cox1 sequences retrieved from Genbank and used in this study.

Country	Animal	Accession number	References
Egypt	Sheep	AB921090	[13]
	Camel	AB921054	
	Camel	AB921055	
	Buffalo	AB921072	
Jordan	Sheep	AB688598	[36]
Iran	Goat	KR337824	Unpublished
	Human	JQ250815	[36]
	Sheep	JQ250816	
	Goat	JQ250807	
	Camel	JQ250814	
	Buffalo	HM130592	[24]
		HM130597	
China	Dog	DQ356882	Unpublished
	Sheep	AB688612	[36]
	Human	AB688613	
		AB688617	
	Cattle	AY389989	Unpublished
	Sheep	AY386210	Unpublished
Tibet plateau	Sheep	KJ628364	Unpublished
Mongolia	Human	AB893250	[37]
	Sheep	AB787532	Unpublished
	Goat	AB787536	
Nepal	Buffalo	AB551110	Unpublished
	Pig	AB522647	Unpublished
Portugal	Sheep	HF947595	[38]
Russia	Human	AB688140	[39]
	Cat	AB622277	[40]
	Human	AB777908	[41]
	Sheep	AB777905	
Peru	Cattle	AB688620	[36]
	Sheep	AB688621	

cox1 = Cytochrome C oxidase subunit 1

**Table-2:** Partial nadh1 sequences retrieved from Genbank and used in this study.

Country	Animal	Accession number	References
Egypt	Sheep	AB921125	[13]
	Camel	AB921091	
	Camel	AB921092	
	Buffalo	AB921109	
Tunisia	Cattle	KT363806	Unpublished
	Cattle	KT005319	
Morocco	Sheep	EF367308	Unpublished
	Cattle	EF367334	
	Camel	EF367316	
Iran	Cattle	HM055619	Unpublished
	Sheep	HM055626	
	Camel	GQ357999	Unpublished
	Goat	KJ162553	Unpublished
India	Buffalo	EF179167	Unpublished
	Sheep	EF179166	
	Buffalo	EF125695	
	Cattle	EF125693	
	Goat	GQ168810	Unpublished
China	Human	KJ556994	[42]
	Cattle	AY386215	Unpublished
	Sheep	AY572546	Unpublished
Tibetan plateau	Sheep	JX217883	[43]
Argentina	Cattle	KC579441	[44]
Peru	Cattle	JF946610	Unpublished

## Results

**Figure-2:**
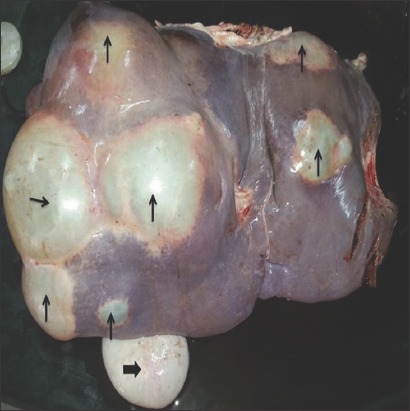
A buffalo’s liver infected with multi-localized large and medium sized hydatid cysts (arrow). The big arrow refers to the gall bladder.

Examination of 120 slaughtered water buffaloes in Mansoura Abattoir, Dakahlia province, Egypt, demonstrated the presence of hydatid cysts in 5 carcasses (4.2%). One infected animal harbored 14 sterile cysts in its liver (Figure-2), while the lung is the affected tissue in four animals (two of them had single fertile cyst infection, one had 2 fertile cysts, and the last one had 9 caseated sterile cysts). Collectively, 4 (14.8%) out of 27 collected cysts were fertile.

### Genotype identification and sequence analysis

Clear and readable nucleotide sequences were revealed from the 5 examined cysts (four fertile lung cysts and one sterile liver cyst). Alignment of the 5 samples with each other indicated that all of them were belonged to the same haplotype, and they were typed under the *E. granulosus* G1 genotype (common sheep strain).

**Figure-3:**
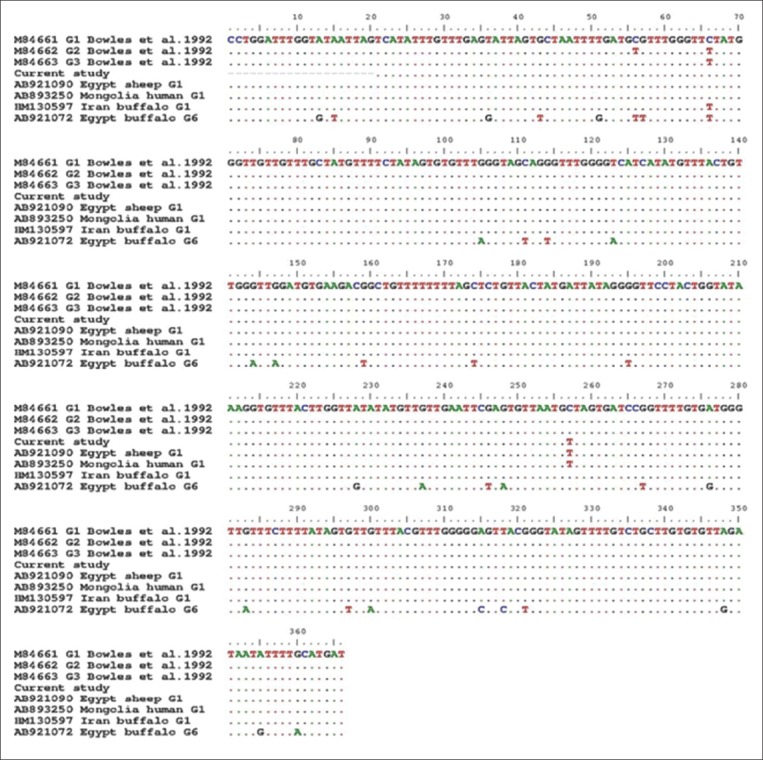
Alignment of partial cytochrome C oxidase subunit 1 sequences of the buffaloes isolate in the present work with variable reported haplotypes on Genbank. This figure shows the complete identity between our G1 haplotype with those reported from the Egyptian sheep (AB921090).

At the level of cox1 gene, single nucleotide substitution (C257T) was noted by alignment of this study haplotype and the reference sequence M84661 (the first described G1 by Bowles *et al*. [14]) (Figure-3). Nevertheless, complete identity was found with a number of G1 isolates from different countries and host species like sheep from Tibet (KJ628364), goats from Iran (KR337824), and human from Mongolia (AB893250).

**Figure-4:**
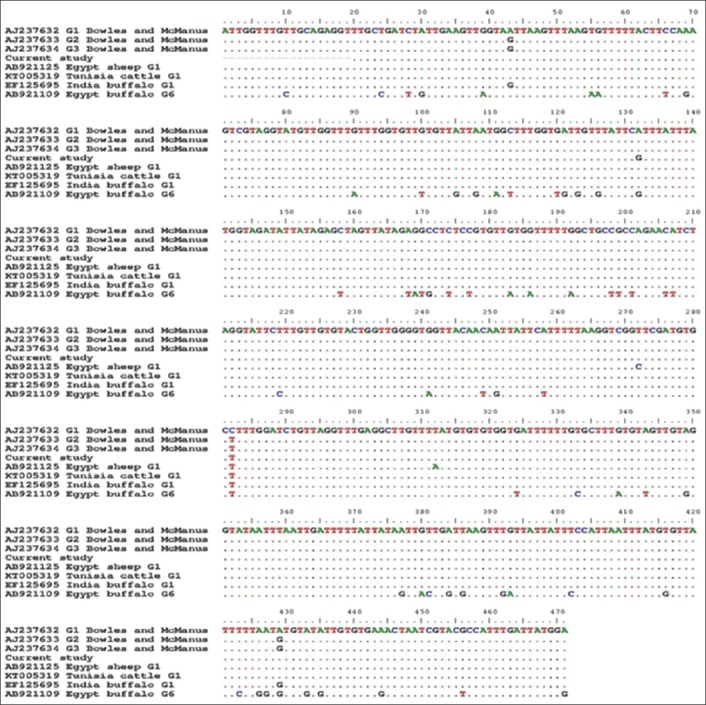
Alignment of partial nicotinamide adenine dinucleotide hydrogen 1 sequences of the buffaloes isolate in the present work with variable reported haplotypes on Genbank. A singleton G1 haplotype was noted in the present work with a unique mutational site (A132G).

Concerning the nadh1 gene, a unique mutational site (A132G) was noted in our haplotype when aligned with different reference sequences (Figure-4), rather than another nucleotide substitution (C282T) with AJ237632 (The first described G1 by Bowles and McManus [15]). Moreover, 99% homology was recorded with the reported G1 genotype from each of Tunisia (KT363806), Morocco (EF367308), Iran (GQ357999), and India (EF179167).

### Phylogenetic analysis

**Figure-5:**
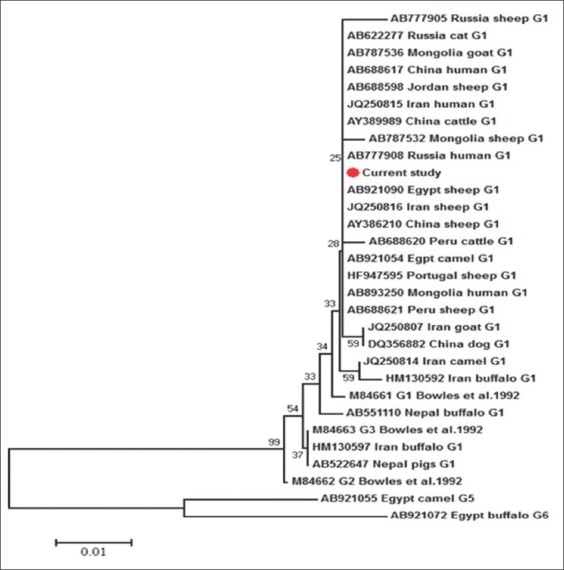
Neighbor-Joining phylogenetic tree of G1 genotype partial cytochrome C oxidase subunit 1 sections from different hosts and geographical regions. G5 and G6 genotypes were used as an out group. The bootstrap analysis was conducted using 1000 replicates. Scale bar indicates the proportion of sites changing along each branch.

**Figure-6:**
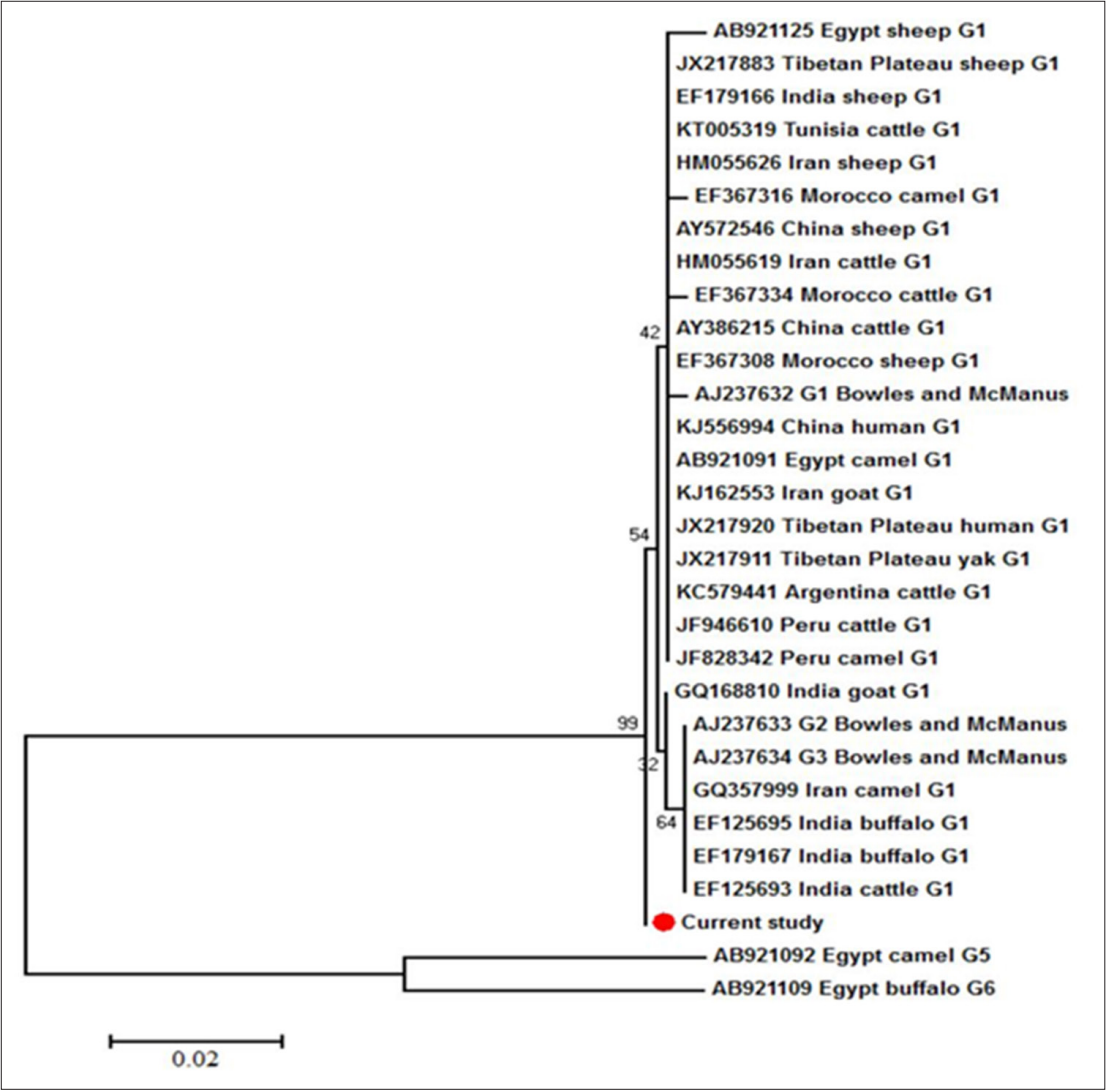
Neighbor-Joining phylogenetic tree of G1 genotype partial nicotinamide adenine dinucleotide hydrogen 1 sections from different hosts and geographical regions. G5 and G6 genotypes were used as an out group. The bootstrap analysis was conducted using 1000 replicates. Scale bar indicates the proportion of sites changing along each branch.

Using the G5 and G6 genotypes from the Egyptian camels and buffaloes, respectively, as an out group, the neighbor-joining phylogenetic trees indicated the clustering of our G1 haplotype within the other G1 haplotypes from variable host species in different geographical regions (Figures-5 and 6). Despite the nadh1 illustrated the separation of our haplotype in a unique branch within the same G1 clade (Figure-6).

## Discussion

CE is an important economic and life-threatening zoonotic disease. In view of its economic importance, we aimed to update the knowledge about CE from water buffaloes slaughtered in Egypt.

In total, the examined 120 buffalo carcasses revealed 4.2% infection with hydatid cysts. The previous report from the same district (Mansoura) recorded 6.4% infection rate [5]. However, three reports from Cairo [16] and Upper Egypt [4,17] did not observe CE infection in the Egyptian buffaloes. This indicates the endemicity of CE in buffaloes at Mansoura, Dakahlia province. Diversified prevalences were reported from different countries such as 42% in Greece [18], 8.7% in Italy [19], 3.81% in India [10], 9% in Iran [20], and 7.19% in Pakistan [21].

Furthermore, lungs appeared to be the predilection site for CE in buffaloes (80% out of the examined animals), coinciding with results from studies conducted in Turkey [8] and Italy [22].

On the other hand, the fertility rate of the harvested cysts was considerably low (14.8%). Our molecular results indicated the homogeneity of those cysts with G1 genotype (common sheep strain). Furthermore, the previous reports discussed the low fertility rate of hydatid cysts of buffaloes (7.3%) and their relation to the G1 [18,20]. While in Pakistan, where the G3 (buffalo strain) was the prevalent genotype, the fertility rate was high (84.3%) [21]. Therefore, this could be explained by the low adaptation of buffaloes to the common sheep strain which increases the tendency of sterile cysts formation [23]. Consequently, our results assumed the reduced role played by buffaloes in maintaining the *Echinococcus* life cycle in this geographical area.

The analyzed sequences of the 5 hydatid cyst isolates in the present work indicated the infection of the Egyptian buffaloes with the G1 genotype. Previously, 2 samples from the Egyptian buffaloes were characterized as G6 which not described before from buffaloes worldwide [13]. Considering reports from buffalo rearing countries (Table-3), G1 was the widely dispersed genotype in Iran [24]; India [25], Pakistan [26], Italy [27], Turkey [8], and Greece [18]. Although, G3 (buffalo strain) and G5 (cattle strain) were recorded but in few cases [7,21,25,28]. This is the first report about the existence of the G1 genotype in buffaloes from Egypt.

**Table-3:** Genotypes distribution of hydatid cyst isolates from water buffaloes within different buffaloes rearing countries.

Country	No.	Genetic marker	Genotype	References
Egypt	2	cox1, nadh1, actinII	G6	[13]
Turkey	9	cox1	G1 (7), G2 (2)	[8]
Greece	24	cox1, nadh1	G1-G3	[18]
Italy	48	cox1	G1 (33), (G2) (15)	[34]
	58	cox1	G1 (35), G2 (8), G3 (15)	[27]
	5	cox1, nadh1	G1 (2), G2 (1), G3 (2)	[7]
	11	12S rRNA	G1 (3), G2 (8)	[22]
Iran	1	cox1	G3	[28]
	25	cox1	G1 (23), G2 (2)	[24]
	4	cox1, nadh1, Atp6, 12S rRNA	G1	[29]
India	6	cox1, nadh1, ITS1	G2	[9]
	7	atp6 and nad2	G1 (6); G5 (1)	[25]
	13	cox1	G1 (3), G3 (8), G5 (2)	[10]
	1	cox1	G3	[35]
Pakistan	4	cox1	G3	[21]
	129	12S rRNA	G1 (76)	[26]

Cox1 = Cytochrome C oxidase subunit 1, nadh1 = Nicotinamide adenine dinucleotide hydrogen subunit 1

The alignment results of the partial cox1 sections (Figure-3) showed the complete matching of the G1 isolates from buffaloes in this work with that of sheep (AB921090) and camels (AB921054) from Egypt, which represented phylogenetically by their sharing of the same branch. On the contrary, our isolates evoked a singleton G1 haplotype, at the level of nadh1 partial sections (Figure-4), which not previously reported from Egypt and worldwide. This haplotype was clustered in a separate branch and nearer to the G1 haplotype from the Indian cattle (EF125693) and buffaloes (EF179167), which assuming the introduction of this haplotype to Egypt through the imported cattle and buffaloes meat from India. Moreover, the previous results illustrated the more usefulness of nadh1 than cox1 in studying *Echinococcus* haplotypes diversity [29], although Bowles and McManus [15] found identical sequences of G2 and G3 for nadh1 gene in the same tested samples with cox1which showed 2 mutational sites between both genotypes (Figure-4).

Little is known about the genotypes of *Echinococcus* from the Egyptian animals and human. Studies illustrated the wide domination of the G6 (camel strain), which favors the role the camel-dog cycle in the transmission of echinococcosis [11,13,30,31]. Here, we have notice that all the collected samples in these studies were originated from the same area (Cairo and Qalubiya provinces), which considered the main localities in Egypt for importing camels and consuming their meats. Furthermore, G1 genotype (common sheep strain) was described in one camel as well as 4 sheep samples [13], and in one out of 31 human samples [11]. In addition, Abd El Baki *et al*. [32] stated that 80% of human and camel isolates were belonged to G1. From the zoonotic point of view, it is well known that the majority of human cysts were typed under the G1 and its variants [1,18,33], and thus emphasis the great role of the sheep-dog cycle in echinococcosis transmission for different reasons, like the wide use of dogs with sheep flocks for protection, the commonness of the out-abattoir sheep slaughter in the rural communities specially in the ceremonies like Al-Adhua feast in Muslims countries and the unsatisfactory offal disposal which enable dogs to access easily the hydatid cysts. This assumption is clearly evident in our results which illustrated the commonness of G1 genotype in buffaloes from Dakahkia province, Egypt.

## Conclusion

In spite of the low infection and fertility rates of buffalo, hydatid cysts recorded in this study, the mission, even a weak, of this animal for keeping G1 genotype life cycle is still important.

## Authors’ Contributions

Study design, samples collection, laboratory work, and the manuscript writing was done by IA. IA has read and approved the final manuscript.
